# Employment status and associated factors in working-age maintenance hemodialysis patients: a multicenter cross-sectional study

**DOI:** 10.1080/0886022X.2026.2630462

**Published:** 2026-03-03

**Authors:** Lan Zhang, Mingcheng Xu, Yanping Peng, Jingxin Zhao, Yancun Li, Fangyong Wang, Yingchun Ma

**Affiliations:** ^a^ School of Rehabilitation, Capital Medical University; China Rehabilitation Research Center, Beijing Boai Hospital, Beijing, China; ^b^ Department of Nephrology, China Rehabilitation Research Center, Beijing Boai Hospital, Beijing, China; ^c^ Hemodialysis Center, Xicheng District Zhanlanlu Hospital, Beijing, China; ^d^ Urology and Metabolism Rehabilitation Center, Beijing Rehabilitation Hospital Affiliated to Capital Medical University, Beijing, China; ^e^ Department of Nephrology, Changping District Integrated Traditional Chinese and Western Medicine Hospital, Beijing, China; ^f^ Department of Nephrology, Daxing District Integrated Traditional Chinese and Western Medicine Hospital, Beijing, China; ^g^ Department of Spine Surgery, Beijing Bo’ai Hospital, China Rehabilitation Research Center, Rehabilitation School of Capital Medical University, Beijing, China; ^h^ Rehabilitation Medicine Center, The Second Affiliated Hospital and Yuying Children’s Hospital, Wenzhou Medical University, Wenzhou, China; ^i^ University of Health and Rehabilitation Sciences, Qingdao, China; ^j^ School of Biological Science and Medical Engineering, Beihang University, Beijing, China; ^k^ Department of Orthopaedics, Qilu Hospital, Cheeloo College of Medicine, Shandong University, Jinan, China; ^l^ School of Rehabilitation Medicine, Shandong University of Traditional Chinese Medicine, Jinan, China

**Keywords:** Maintenance hemodialysis, employment status, workforce participation, body mass index, vocational rehabilitation

## Abstract

**Objectives:**

To investigate the employment status and analyze its associated factors among working-age patients undergoing maintenance hemodialysis (MHD).

**Methods:**

This multicenter, cross-sectional study enrolled 126 working-age (18–60 years) MHD patients from five dialysis centers in Beijing between January 2023 and January 2024. Baseline characteristics, psychological status (using Beck Depression Inventory and State-Trait Anxiety Inventory), and clinical data were collected via face-to-face questionnaires during dialysis sessions. Participants were categorized into employed and non-employed groups based on current work status.

**Results:**

Among the 126 patients, 33.3% (*n* = 42) were employed, 38.1% stopped working due to illness, and 26.2% of non-working patients expressed willingness to return to work if given the opportunity. Occupations among employed patients were primarily company employees (33.3%) and self-employed/freelancers (40.5%). Multivariate logistic regression identified female sex (OR = 0.166, 95% CI: 0.053–0.515, *p* = 0.002), older age (OR = 0.895, 95% CI: 0.847–0.947, *p* < 0.001), and higher annual household income (OR = 0.328, 95% CI: 0.196–0.549, *p* < 0.001) as independent factors negatively associated with employment, while higher body mass index (BMI) was positively associated with employment (OR = 1.362, 95% CI: 1.025–1.810, *p* = 0.033).

**Conclusions:**

Approximately, one-third of working-age MHD patients in Beijing remain employed, with flexible work forms (self-employment/freelancing) being common. Female gender, older age, and higher household income are significant negative correlates of employment, whereas higher BMI is positively associated with employment. These findings highlight the importance of nutritional status and suggest the need for targeted vocational rehabilitation strategies to improve workforce participation and social reintegration in this population.

Maintenance hemodialysis (MHD) is one of the most common renal replacement therapies for patients with end-stage renal disease (ESRD). According to the Chinese National Renal Data System (CNRDS), by the end of 2024, there were approximately 1.027 million MHD patients in mainland China, with 196,726 new patients added in 2024, indicating a rapidly increasing prevalence and growth rate. Hemodialysis treatment should not signify the end of a patient’s social and occupational life [1]. Advances in medicine mean that most MHD patients, with adequate dialysis, see corrections of complications like renal anemia and metabolic acidosis. Interventions such as exercise rehabilitation [2–4] and psychological rehabilitation [5,6] improve activities of daily living and quality of life, enabling patients to continue working, traveling, exercising, and engaging in social activities while receiving treatment. Investigating employment status and its influencing factors is crucial for facilitating the reintegration of MHD patients into society and helping them realize their social value.

This study aims to survey the employment status of MHD patients across multiple dialysis centers in Beijing and analyze the related influencing factors.

## 1. Materials and methods

### 1.1. Study participants

MHD patients were recruited from five hemodialysis centers in Beijing (Beijing Bo’ai Hospital, China Rehabilitation Research Center, Xicheng District Zhanlanlu Hospital, Beijing Rehabilitation Hospital affiliated to Capital Medical University, Changping District Integrated Traditional Chinese and Western Medicine Hospital, and Daxing District Integrated Traditional Chinese and Western Medicine Hospital) between January 2023 and January 2024. Inclusion criteria were: (1) undergoing regular hemodialysis for ≥3 months; (2) within the legal working age (defined as ≥18 and ≤60 years old in this study, considering varying retirement ages in China); and (3) voluntary participation with signed informed consent. Exclusion criteria were: (1) history of surgery, trauma, severe infection, or diagnosed malignancy within the previous 3 months; (2) poor vision or hearing; (3) limb amputation; and (4) communication barriers. This study was approved by the institutional ethics committee. Employment was defined as engaging in activities for remuneration or profit within the legal working age.

Based on the empirical rule in statistics requiring 10–15 times the number of independent variables, and with 10 planned variables, a minimum sample size of 100 patients was targeted.

### 1.2. Survey method

A cross-sectional survey was conducted using questionnaires administered face-to-face during dialysis sessions. Completed questionnaires were submitted electronically.

### 1.3. Survey tools

(1) An electronic questionnaire was designed using Wenjuanxing (Questionnaire Star). The questionnaire, integrated from previous studies, included baseline data: sex, age, BMI, current employment status, primary renal disease, dialysis vintage, education level, annual household income, and payment method (categorized based on literature [7,8]). (2) The Beck Depression Inventory (BDI), which has 91% sensitivity and 96% specificity for detecting depression [9]. (3) The State-Trait Anxiety Inventory (STAI) for assessing anxiety [10].

Clinical data from the long interdialytic interval, including urea clearance index (*Kt*/*V*), serum albumin, intact parathyroid hormone (iPTH), were collected from the hospital information system. Participants were divided into employed and non-employed groups based on work status.

### 1.4. Data screening

Data exported from the platform were checked for outliers and duplicates. One researcher organized the data, and another verified it independently to ensure accuracy.

### 1.5. Quality control

Quality control measures included prescreening participants, setting reasonable questionnaire completion times, and checking for duplicate responses.

### 1.6. Statistical analysis

Data were analyzed using SPSS Statistics software (version 22.0) (Chicago, IL). Normally distributed continuous data were expressed as mean ± standard deviation (SD); non-normally distributed data were expressed as median (interquartile range, IQR). Categorical data were expressed as numbers and percentages. Differences between the employed and non-employed groups were compared using Student’s *t*-test (normal continuous data), Chi-square test (categorical data), or Wilcoxon’s rank-sum test (ordinal data). Variables with *p* < 0.20 in univariate analysis were included in a multivariate logistic regression model to identify independent factors influencing employment status, expressed as odds ratios (ORs) with 95% confidence intervals (CIs). A stepwise forward method was used for variable selection, with a two-sided significance level of *α* = 0.05.

## 2. Results

### 2.1. General characteristics

All 126 distributed questionnaires were returned and deemed valid. The study included 126 MHD patients (85 males, 41 females). Forty-two patients were employed (29 full-time, 13 part-time), and 84 were not employed (12 normal retirement, 39 medical retirement, nine resigned due to illness, 24 unemployed). Univariate analysis (Table 1) showed significant differences between groups in gender, age, serum albumin, annual household income, education level, and body mass index (BMI) (*p* < 0.05). No significant differences were found in other indicators (*p* > 0.05).

**Table 1.** Univariate analysis of factors associated with employment in MHD patients (*n* = 126).

Item	Employed group (*n* = 42)	Non-employed group (*n* = 84)	Statistic	*p* Value
Sex (male/female, *n* (%))	35/7	50/34	*χ*^2^ = 7.231	0.007
Age (years, mean ± SD)	43.9 ± 8.6	50.6 ± 8.3	*t* = 4.273	<0.001
Dialysis vintage (months, median (IQR))	44.0(28.5, 71.5)	53.0 (26.0, 77.5)	*Z* = −1.302	0.193
Hemoglobin (g/L, mean ± SD)	111.7 ± 11.5	112.3 ± 12.7	*t* = 0.250	0.803
Albumin (g/L, mean ± SD)	40.2 ± 2.9	39.0 ± 3.1	*t* = −2.024	0.046
BMI (kg/m^2^, mean ± SD)	26.0 ± 6.1	23.9 ± 4.3	*t* = −2.211	0.029
*Kt*/*V* (mean ± SD)	1.3 ± 0.3	1.4 ± 0.3	*t* = 1.122	0.265
Primary disease, *n*				
Diabetic nephropathy	18	36	*χ*^2^ = 18.926	0.462
Hypertensive nephropathy	5	18		
Other secondary glomerulonephritis	4	3		
Chronic glomerulonephritis	12	14		
Cystic kidney disease	3	11		
Tubulointerstitial disease	0	1		
Other/unknown	0	1		
Annual household income, *n*				
≤¥50,000	25	19	*Z* = −4.233	0.000
>¥50,000 and ≤¥100,000	9	24		
>¥100,000 and ≤¥200,000	6	19		
>¥200,000	2	22		
Education level, *n*				
Junior high school or below	6	2	*Z* = −3.040	0.002
High school	4	13		
College	19	18		
Bachelor’s degree	10	30		
Master’s degree or above	3	21		
Payment method, *n*				
Beijing medical insurance	42	82	*χ*^2^ = 2.077	0.557
Other insurance/self-pay	0	2		
STAI-state score (mean ± SD)	40.2 ± 10.8	42.1 ± 10.8	*t* = 0.902	0.370
STAI-trait score (mean ± SD)	40.7 ± 9.9	42.6 ± 9.5	*t* = 0.978	0.331
BDI score (mean ± SD)	22.9 ± 11.2	25.4 ± 15.1	*t* = 1.064	0.290

### 2.2. Occupational distribution

Among the 42 employed patients, occupations were: worker (3, 7.1%), farmer (1, 2.4%), company employee (14, 33.3%), public institution employee (6, 14.3%), self-employed (12, 28.6%), freelancer (5, 11.9%), and other (1, 2.4%).

### 2.3. Multivariate logistic regression analysis of factors influencing employment

Variables with *p* < 0.20 in univariate analysis (gender, age, dialysis vintage, albumin, annual household income, education level, BMI) were included in the multivariate logistic regression model (variable assignments in Table 2). The results (Table 3) identified female gender, older age, and higher annual household income as factors negatively associated with employment, and identified higher BMI as a factor positively associated with employment (*p* < 0.05). Dialysis vintage, albumin, and education level were not retained in the final model. The results showed that the Hosmer–Lemeshow test statistic was *χ*^2^ = 1.745, df = 8, *p* = 0.988 and the AUC was 0.858 (95% CI: (0.790–0.925))

**Table 2.** Variable assignments.

Variable	Assignment
Dependent variable	
Employment status	No = 0; yes = 1
Independent variables	
Sex	Male = 0; female = 1
Annual household income	≤¥50,000 = 0; >¥50,000 and ≤¥100,000 = 1; >¥100,000 and ≤¥200,000 = 2; >¥200,000 = 3
Education level	Junior high/below = 0; high school = 1; college = 2; bachelor’s = 3; master’s+ = 4
Age	Continuous
Dialysis vintage	Continuous
Albumin	Continuous
BMI	Continuous

**Table 3.** Multivariate logistic regression analysis of factors influencing employment (*n* = 126).

Variable	*β*	SE	Wald *χ*^2^	OR	95% CI for OR	*p* Value
Sex (female)	−1.797	0.578	9.655	0.166	(0.053–0.515)	0.002
Age	−0.110	0.028	15.119	0.895	(0.847–0.947)	0.000
Annual household income	−1.114	0.263	18.023	0.328	(0.196–0.549)	0.000
BMI	0.309	0.145	4.525	1.362	(1.025–1.810)	0.033

Hosmer–Lemeshow test: *χ*^2^ = 1.745, df = 8, *p* = 0.988.

## 3. Discussion

MHD is a primary life-sustaining treatment for ESRD patients. However, the demanding treatment schedule and associated complications often lead to declines in physical and psychological function [11,12], causing fatigue, anxiety, and depression [13,14], reducing social participation, and consequently affecting employment [15]. Reported employment rates among dialysis patients vary between 10% and 44% depending on the population studied [7,8]. The fixed schedule of in-center hemodialysis often conflicts with standard working hours, significantly limiting job opportunities for MHD patients. This constraint mirrors a central issue in occupational health: the conflict between rigid scheduling demands and sustainable employment. The literature on shift work extensively documents how fixed, nonstandard hours can limit job opportunities, affect well-being, and require specific workplace accommodations—challenges that are directly analogous to those faced by MHD patients bound to dialysis schedules. A study from Huashan Hospital, Fudan University, in Shanghai reported an employment rate of only 15.7% among 231 MHD patients [16]. In this study, the employment rate among MHD patients was 33.3%, higher than the rate reported in the aforementioned research. This discrepancy may be attributed to the fact that all dialysis centers selected for this study are located in Beijing. As a first-tier city, Beijing offers more employment opportunities compared to other cities. Furthermore, traditional employment is often constrained by workplace location, working hours, and specific job requirements for practitioners. With social progress and the continuous development of the internet, current forms of employment in society have diversified. Emerging work models such as e-commerce, ride-hailing services, and live-streaming have proliferated. Consequently, MHD patients have the opportunity to choose work with flexible hours and high adaptability, allowing them to better coordinate the conflict between work and hemodialysis schedules. This study shows that the combined proportion of self-employed individuals and freelancers is as high as 40.5%, significantly exceeding the proportion of traditional occupations, reflecting this trend to some extent.

A considerable proportion (38.1%) of patients stopped working due to illness (medical retirement or resignation), highlighting the impact of ESRD and dialysis on workforce participation.

Factors influencing employment can be subjective (patient willingness) and objective (external barriers). Domestically, one study found only 34% of unemployed MHD patients desired to work [16]. In our study, 11.1% of capable patients were unwilling to work, and only 26.2% of non-working patients expressed willingness to return to work, indicating generally low motivation, possibly due to disease-related concerns and low self-efficacy. Notably, our analysis revealed a patient subgroup possessing what can be termed ‘latent employability’ – those who are physically capable of working and express willingness to work but remain unemployed (comprising 11.1% of capable patients unwilling to work and 7.9% capable but unemployed). This conceptual distinction identifies an invisible yet crucial target population for intervention, differentiating our study from purely descriptive surveys. Patients with latent employability represent the most promising candidates for vocational rehabilitation programs, as they already possess both the physical capacity and motivational foundation for employment. Targeted interventions for this group – such as workplace accommodation guidance, skills training, and job matching services – could yield substantial returns in improving overall employment rates. This finding underscores that unemployment in MHD patients is not merely a binary outcome but exists along a continuum where psychological, social, and systemic barriers may prevent capable individuals from entering or returning to the workforce.

It is noteworthy that anxiety and depression scores did not show significant group differences in univariate analysis and were not retained in the final multivariate model. This could partly be attributed to the mediating or confounding effects of the demographic and clinical factors included in our model (e.g., age, income, BMI). Additionally, the relatively modest sample size of this study may have limited the statistical power to detect the potentially subtle or nonlinear effects of these complex psychosocial constructs on employment status. The psychological burden of a lifelong, debilitating disease requiring a demanding treatment regimen is immense. Symptoms of depression, such as anhedonia, fatigue, and low motivation, can directly diminish the desire and capacity to seek or maintain employment [17]. Similarly, anxiety, including concerns about health stability, ability to perform tasks, and social interactions in the workplace, can act as a significant psychological barrier [18]. Therefore, the non-significance in our model should not be interpreted as evidence against the importance of mental health in vocational outcomes. Future investigations with larger, well-powered cohorts and more nuanced psychological assessments are warranted to clarify the specific role and mechanism of anxiety and depression in employment among MHD patients.

Objective factors include dialysis schedule, comorbidities, psychological state, socioeconomic status, and nutritional status. Huang et al. [16] linked lower employment to older age, lower education, and surprisingly, higher family income. Our study confirms older age and higher family income as negative correlates and newly identifies female gender as a significant independent negative factor. The inverse association between higher household income and employment may reflect greater family-level financial buffering, which reduces the economic necessity for the patient to remain employed, rather than indicating a lower individual earning capacity. This might be particularly relevant in urban Chinese settings where family support networks are strong. Preexisting gender biases in the job market, potentially exacerbated by policies like the expanded birth policy and concerns about reliability due to health issues, likely contribute to this disparity.

Notably, we identified BMI as an independent influencing factor, with each unit increase (kg/m^2^) associated with a 1.362 times higher odds of employment. Thus, a higher BMI was positively associated with employment status in our cohort. This underscores the importance of nutritional status, a novel finding in the Chinese context. Protein-energy wasting is a known risk factor for mortality in MHD patients [19,20]. Malnutrition leads to sarcopenia, anemia, and infections, causing frailty and severely impairing quality of life [21,22], inevitably affecting employability. Dialysis centers should prioritize nutritional management, adequate dialysis, complication correction, dietary guidance, and, where possible, exercise rehabilitation programs to improve muscle strength and physical function, thereby potentially improving nutritional markers and employment prospects. Our previous research showed that intradialytic aerobic combined with resistance exercise improved nutritional parameters [23]. However, BMI should be maintained within an optimal range (e.g., not exceeding 24 kg/m^2^) to avoid risks associated with overweight/obesity [24,25].

It is important to note that the observed positive association between BMI and employment status in this cross-sectional study does not confirm a causal direction. While we hypothesize that better nutritional status (reflected by a higher BMI within a healthy range) may enhance physical capacity and thus employability, an equally plausible explanation is reverse causation. Employment itself could positively influence nutritional status and BMI through several pathways. A stable job often provides better financial resources, enabling improved access to a variety of nutritious foods and potentially more balanced diets. Furthermore, employment confers structure to daily life, social engagement, and a sense of purpose, which may collectively contribute to better overall well-being and health-maintaining behaviors, including dietary habits. The psychological and economic benefits of being employed might also mitigate disease-related anorexia or malnutrition. Therefore, the relationship between BMI and employment in MHD patients is likely bidirectional and complex. Our findings highlight a significant association warranting attention, but longitudinal studies are necessary to disentangle the temporal sequence and establish whether improving nutritional status can indeed lead to better employment outcomes, or if facilitating employment is a key intervention to improve the nutritional health of this vulnerable population.

Contrary to some studies [7,8,16], education level and payment method were not significant factors in our multivariate model. This might be due to the specific characteristics of our cohort; 80.2% had college education or higher, and Beijing has high medical insurance coverage, potentially introducing selection bias.

### 3.1. Limitations

First, the sample was drawn exclusively from five dialysis centers in Beijing. As a major metropolitan area, Beijing’s healthcare resources, socioeconomic conditions, and employment landscape may differ systematically from other regions in China. Consequently, our sample may not be fully representative of the broader MHD patient population nationwide, particularly those in rural or economically underdeveloped areas. Second, the requirement for participants to complete the questionnaire may have excluded patients with severe cognitive impairment, visual/hearing deficits, or very low literacy, potentially biasing the sample toward individuals with better overall functional and cognitive status. Third, these geographical and enrollment criteria limitations jointly affect the external validity of our findings, and caution should be exercised when generalizing the conclusions to the wider MHD population. Fourth, the cross-sectional design precludes the establishment of causal relationships. Fifth, this study defined employment status as a binary variable (employed/unemployed) and did not investigate specific dimensions of work, such as job stability (e.g., full-time/part-time, type of contract), income level, working hours, and the compatibility of work with dialysis schedules. These dimensions are crucial for a deeper understanding of the quality of employment, economic independence, and the actual degree of socioeconomic reintegration among MHD patients. While our current findings primarily reflect the ‘likelihood’ of employment, future research should aim to assess the ‘quality’ and ‘sustainability’ of employment.

### 3.2. Recommendations for practice

Our findings underscore the need for innovative and accessible support strategies. In this context, we propose a pragmatic approach: embedding vocational rehabilitation services within the dialysis unit setting. Hemodialysis treatment requires patients to remain in the clinic for approximately 4 h, three times per week, creating a substantial yet often passive block of time. This intradialytic period could be transformed into an active resource for delivering structured vocational support. Interventions could include, but are not limited to, digital literacy training, guidance on identifying and applying for flexible/remote jobs, counseling on workplace disclosure and accommodations, and even facilitated remote work trials using provided devices. This model offers several advantages: it eliminates separate travel and time commitments for rehabilitation, leverages the existing healthcare relationship and safe environment, and allows for peer support and group-based activities. By integrating vocational goals into routine care, dialysis centers can move beyond purely medical management toward holistic support that addresses patients’ social and economic well-being.

### 3.3. Future research directions

Future directions of study should prioritize longitudinal and multi-regional research designs to clarify causal relationships between demographic, clinical, psychosocial, and socioeconomic determinants and employment outcomes among MHD patients, extending beyond the limitations of cross-sectional analyses. Future studies should integrate larger, nationally representative cohorts and include underexplored variables such as workplace accommodations, flexible dialysis modalities (e.g., home hemodialysis or nocturnal dialysis), employer attitudes, labor policies, and digital or remote employment opportunities. In addition, mechanistic investigations examining how nutritional status, physical function, and mental health interact to influence work capacity are warranted, ideally through interventional trials incorporating structured exercise rehabilitation, nutritional optimization, and psychological support. Mixed-methods approaches combining quantitative modeling with qualitative interviews could further elucidate patient motivation, perceived barriers, and facilitators to reemployment. Collectively, these directions would support the development of targeted vocational rehabilitation programs and evidence-based policy interventions aimed at improving social reintegration and quality of life for working-age MHD patients.

In conclusions, employment rates for MHD patients may be improving with diversifying job markets. This study found an employment rate of 33.3%. Female gender, older age, and higher household income were independent negative factors associated with employment, while higher BMI was positively associated with employment. The association with BMI highlights the critical role of nutritional status. Dialysis centers should focus on optimizing nutrition and physical condition to enhance employability and support patients’ social reintegration.

## Data Availability

The authors confirm that the data supporting the findings of this study are available within the article and its supplementary materials.
